# Effects of Different Viscous Guar Gums on Growth, Apparent Nutrient Digestibility, Intestinal Development and Morphology in Juvenile Largemouth Bass, *Micropterus salmoides*

**DOI:** 10.3389/fphys.2022.927819

**Published:** 2022-08-05

**Authors:** Yu Liu, Yumeng Zhang, Jiongting Fan, Hang Zhou, Huajing Huang, Yixiong Cao, Wen Jiang, Wei Zhang, Junming Deng, Beiping Tan

**Affiliations:** ^1^ College of Fisheries, Guangdong Ocean University, Zhanjiang, China; ^2^ Aquatic Animals Precision Nutrition and High-Efficiency Feed Engineering Research Centre of Guangdong Province, Zhanjiang, China; ^3^ Key Laboratory of Aquatic, Livestock and Poultry Feed Science and Technology in South China, Ministry of Agriculture, Zhanjiang, China

**Keywords:** guar gum, viscous, growth performance, apparent digestibility, intestinal morphology, *Micropterus salmoides*

## Abstract

An 8-week feeding trial was conducted to investigate the effects of different viscous guar gums on the growth performance, apparent nutrient digestibility, intestinal development and morphology of juvenile largemouth bass. Four isoproteic and isolipidic diets (crude protein 42.5%, crude lipid 13.7%) were formulated to contain 8% cellulose (Control group), 8% low viscous guar gum with 2,500 mPa s (Lvs-GG group), 8% medium viscous guar gum with 5,200 mPa s (Mvs-GG group) and 8% high viscous guar gum with 6,000 mPa s (Hvs-GG group), respectively. Each diet was fed to quadruplicate groups of 40 fish (6.00 ± 0.01 g) per repetition. Dietary guar gum inclusion significantly decreased the weight gain rate, specific growth rate, protein efficiency ratio, protein productive value and lipid deposition rate, and these parameters decreased considerably with increasing guar gum viscous and were lowest in the Hvs-GG group. Dietary guar gum inclusion significantly decreased the apparent digestibility of dry matter, crude protein and crude lipid, and these parameters decreased considerably with increasing guar gum viscous and were lowest in the Hvs-GG group. Intestinal protease, lipase and creatine kinase activities in the guar gum groups were significantly lower than those in the control group, and intestinal protease and lipase activities decreased considerably with increased guar gum viscous. Intestinal alkaline phosphatase activity in the Hvs-GG group and intestinal Na^+^/K^+^-ATPase activity in the Mvs-GG and Hvs-GG groups were significantly lower than those in the Lvs-GG and control groups. Serum high-density lipoprotein, total cholesterol and triglyceride concentrations and superoxide dismutase activity in the guar gum groups were significantly lower than those in the control group. Intestinal villus height and muscular thickness in the guar gum groups were considerably higher than those in the control group, whereas the goblet cell relative number in the Mvs-GG and Hvs-GG groups and the microvillus height in the Lvs-GG and Hvs-GG groups were significantly lower than those in the control group. The expression level of IGF-1 in the guar gum groups and the expression level of GLP-2 in the Mvs-GG and Hvs-GG groups were significantly higher than those in the control group. These results indicated that guar gum diets adversely affected intestinal morphology, decreased intestinal digestive and absorptive enzyme activities, and caused poor nutrient digestibility and growth performance in juvenile largemouth bass. Moreover, the adverse effects of guar gum are closely related to its viscous, and high viscous guar gum produces more extreme negative impacts on juvenile largemouth bass.

## Introduction

Limited fishmeal production and a rapid development of aquaculture have resulted in increasingly high fishmeal prices, which has largely increased farming costs and limited the further development of aquaculture (Yannis et al., 2018; Zhang et al., 2018; Zhang et al., 2019; Yin et al., 2021). The search for feasible alternatives to fishmeal has attracted widespread attention from researchers and considerable progress has been made in the last few decades. Multiple protein sources, including plant-based proteins, animal by-products and single-cell proteins have been tested as feasible alternatives to fishmeal (Zeng et al., 2018). In particular, plant proteins are considered the most promising fishmeal substitutes due to their wide availability, high yield, stable quality and low price (Deng et al., 2017). However, the presence of anti-nutritional factors (ANFs) limited the use of plant proteins in aquafeeds (Collins et al., 2013; Azeredo et al., 2017). ANFs, such as non-starch polysaccharides (NSPs), trypsin inhibitors, antigenic proteins and saponins have been shown to be detrimental to fish growth and health (Takii et al., 2008; Merrifield et al., 2009; He et al., 2020; Deng et al., 2021). NSPs are major components of the plant cell wall, and are abundant in plant feed ingredients (10–70%, varies with plant species), including cellulose, hemi-cellulose and pectin, which can be divided into insoluble and soluble NSPs (Sinha et al., 2011; Choct, 2015). Therefore, the use of plant proteins in aquafeeds increases the level of NSPs in fish diets (Deng et al., 2021). Fish cannot directly utilize dietary NSPs due to the lack of endogenous NSPs-degrading enzymes (Kuz Mina, 1996); thus, dietary NSPs usually remain in the digestive tract and produce different physiological effects. In recent years, an increasing number of studies have begun to investigate the physiological effects of dietary NSPs on fish (Amirkolaie et al., 2005; Kraugerud et al., 2007; Glencross et al., 2012; Cai et al., 2019; Ren et al., 2020; Deng et al., 2021), and these studies showed that dietary NSPs exert beneficial or detrimental physiological effects on fish were type-dependent, due to the difference in their physicochemical characteristics. Generally, SNSP is considered to have more substantial anti-nutritional effects than INSP, and high-dose dietary SNSP even impairs intestinal or hepatic health in fish (Sinha et al., 2011; Cai et al., 2019; Deng et al., 2021). Studies have also indicated that the adverse effects of dietary SNSP mainly derived from its viscosity (Sinha et al., 2011; Cai et al., 2019; Deng et al., 2021), but few studies have covered this issue.

Guar gum is mainly galactomannan (hemi-cellulose), a viscous SNSP derived from the seeds of *Cyamopsis tetragonolobus* with massive yield and excellent bonding properties (Kazuhiko et al., 1993; Yu et al., 2015). Moreover, the bonding properties (viscosity) of guar gum can be regulated by modifying pH-responsive functional groups such as -CH_3_, -COOH, -SO_3_H, and -CONH_2_ (George et al., 2019). Nowadays, guar gum is widely used as a stabilizer and thickener in food, pharmaceutical and cosmetic processing to improve product quality (Theocharidou et al., 2022). More recently, guar gum has attracted increasing attention as a feasible aquafeeds binder due to its binding properties, showing the ability to improve feed quality (water stability, hardness), reducing nutrient loss and water pollution (Thombare et al., 2016; Gao et al., 2019; Karim et al., 2022). Moreover, dietary guar gum can produce various physiological effects on animals. In mammals, guar gum has been reported to increase digesta viscosity and short-chain fatty acid concentration, reduce serum cholesterol and fecal pH (Jenkins et al., 1975; Kazuhiko et al., 1993; Seal and Mathers, 2001). In fish, studies have shown that dietary guar gum decrease nutrient digestibility and fish growth (Storebakken, 1985; Amirkolaie et al., 2005; Ramos et al., 2015; Gao et al., 2019). Some of these studies pointed out that dietary guar gum exerts beneficial or detrimental effects on fish growth was dose-dependent, dietary supplementation with high doses of guar gum generally exhibiting inhibitory effects on fish growth (Ramos et al., 2015; Gao et al., 2019). Increasing dietary guar gum levels leads to an increase in dietary viscosity (Casas et al., 2000; Ramos et al., 2015), so it can be assumed that the adverse effects of high doses of guar gum on fish growth may be induced by high viscosity. However, the association between guar gum viscosity and its anti-nutritional effect remains unclear.

Largemouth bass *Micropterus salmoides*, a typical freshwater carnivorous fish that is widely cultured due to its strong adaptability and fast growth rate (Li et al., 2007; Li et al., 2010; Hu et al., 2019; Liu et al., 2022). In 2019, the cultural production of this species in China reached 477,808 tons (Xu et al., 2022), which generate considerable economic benefits. With aquaculture development, more plant ingredients will inevitably be used in aquafeeds (Kokou and Fountoulaki, 2018), which poses more severe dietary NSPs challenges for farmed fish. The natural diet of carnivorous fish does not contain NSPs, so that dietary NSPs may have more extreme physiological effects on carnivorous fish. However, there are limited studies related to dietary NSPs on carnivorous fish, including largemouth bass. Therefore, this study will investigate the effects of different viscous guar gum on growth, apparent nutrient digestibility, intestinal development and morphology in juvenile largemouth bass, so as to reveal the association between guar gum viscosity and its anti-nutritional effects in carnivorous fish. Moreover, these results will provide data support for the application of guar gum as a binder for aquafeed in the future.

## Materials and Methods

### Experimental Diets

Four isoproteic and isolipidic diets were formulated (crude protein 42.5%, crude lipid 13.7%) to contain 8% cellulose (Control group), 8% low viscous guar gum with 2,500 mPa s (Lvs-GG group), 8% middle viscous guar gum with 5,200 mPa s (Mvs-GG group) and 8% high viscous guar gum with 6,000 mPa s (Hvs-GG group), respectively. All ingredients were ground into powder and passed through a 0.30 mm diameter sieve, then accurately weighed and mixed evenly with a Hobart-type mixer (JS-14, Zhejiang Zhengtai Elecric Co., Ltd., China). Then, fish oil, soybean oil, soybean lecithin and pure water were added to make a dough. The dough was extruded through a 2.0 mm diameter die using a double-helix extruder (F-75, South China University of Technology, China) to obtain experimental feeds. Finally, the feeds were air-dried at room temperature and stored at -20°C until used. The formulation and proximate compositions of experimental diets are shown in Table 1.

**TABLE 1:** The formulation and approximately composition of experimental diets (%).

Group	C	Lvs-GG	Mvs-GG	Hvs-GG
Ingredients				
Fish meal^a^	45.00	45.00	45.00	45.00
Corn gluten meal	10.00	10.00	10.00	10.00
Soy protein isolate	15.00	15.00	15.00	15.00
Fish oil	4.50	4.50	4.50	4.50
Soy oil	3.40	3.40	3.40	3.40
Soy lecithin	1.00	1.00	1.00	1.00
Starch	10.00	10.00	10.00	10.00
Cellulose	8.00			
Lvs-GG^b^		8.00		
Mvs-GG^b^			8.00	
Hvs-GG^b^				8.00
Ca(H_2_PO_4_)_2_	1.00	1.00	1.00	1.00
NaCl	0.20	0.20	0.20	0.20
Choline chloride	0.30	0.30	0.30	0.30
Vitamin C	0.03	0.03	0.03	0.03
Vitamin and Mineral premix^c^	1.50	1.50	1.50	1.50
Ethoxyquin^a^	0.02	0.02	0.02	0.02
Yttrium (III) oxide	0.05	0.05	0.05	0.05
Proximate composition				
Moisture	10.69	10.76	10.73	10.80
Crude protein	42.59	42.53	42.50	42.54
Crude lipid	13.75	13.70	13.69	13.72
Ash	9.70	9.62	9.68	9.71
Viscosity (mPa•s)	5.14	102.26	221.75	385.80

a Supplied by Zhanjiang Haibao Feed Co., Ltd. (Zhanjiang, China); fish meal, 65.81% crude protein, 7.69% crude lipid.

b Supplied by Guangrao Liuhe Chemical Co., Ltd. (Dongying, China).

c Supplied by Qingdao Master Biotech (Qingdao, China).

### Fish and Feeding Trial

Largemouth bass juveniles were obtained from the freshwater aquaculture base of Guangdong Ocean University. Largemouth bass juveniles have fasted for 24 h before grouping, and 640 individuals with a healthy physique, no disease or injury and a uniform size (6.00 ± 0.01 g) were selected as experimental fish. These fish were randomly divided into four groups with four replicates per group, and each net cage had 40 fish. All net cages have a uniform size (1.2 m × 0.8 m × 1.0 m) and they are placed in the same cement pool. During the feeding period, the water temperature was controlled at 28–31°C with ammonia nitrogen <0.02 mg/L through adjusting flow rate and water change, and the dissolved oxygen was controlled at > 6.0 mg/L by continuous oxygenation with an oxygenator (SC-150DX, Ningbo Yinzhou Hengxi Saier Electric Factory, China). The feeding trial lasted for 8 weeks, and the test fish were fed twice daily until they were satiated (07:00 and 17:00). The number and body weight of fish mortalities and feed consumption in each cage were recorded.

### Digestibility Trial

Digestibility trial was performed during the feeding trial according to the method described by He et al. (2021). Briefly, yttrium trioxide (99.9%, Sinopharm Chemical Reagent Co., Ltd., Shanghai, China) was used as an indicator in the experimental diets. Fecal collection was performed after the test fish had been accustomed to the experimental diets for 2 weeks. Specifically, feces were collected daily from the bottom of the net cages using a 200-mesh brail after the test fish had been fed for 5–7 h. Subsequently, complete feces were selected and dried at 65°C for 6 h, and then stored at −20°C for until use. Fecal collection lasts for 6 weeks to ensure that the fecal samples meet the test requirements. The determination of yttrium content in fecal and diets was carried out by inductively coupled plasma mass spectrometry Briefly, a 100–200 mg sample was first digested by adding 6 ml of nitric acid and 1 ml of hydrogen peroxide with a microwave digestion apparatus (Anton Paar Multiwave PRO 41HVT56, Austria), and the digested solution was subsequently taken for determination using a mass spectrometry (Agilent 7500cx, United States).

### Sampling Strategy

At the end of the feeding trial, fish were fasted for 24 h. The ultimate fish in each net cage was accurately weighed and counted to calculate the growth indices. Before sampling, the test fish were anesthetized with 0.1 g/L of MS-222 (Sigma, United States). Four fish were randomly selected from each cage, and the total length of the fish was measured, weighed and then dissected; the visceral mass, liver and intestine were sequentially weighed, and the length of the intestine was measured. Another four fish were randomly selected from each cage, and the blood was drawn from the tail vein using a 1-ml syringe. The blood samples were transferred to EP tubes, left at 4°C overnight and centrifuged at 3,500 r/min for 10 min. Finally, the supernatant was taken as a serum sample and stored at −80°C for subsequent analysis. Another two fish were randomly selected from each cage for dissection, and their intestines were taken out and placed in EP tubes with RNAlater, snap-frozen in liquid nitrogen and then stored at −80°C for subsequent analysis. Another three fish were randomly selected from each cage and frozen at −20°C for whole-body composition analysis.

### Morphological Observation of Hindgut

One fish was randomly selected from each cage for dissection; the hindgut (1 cm) was taken out and fixed with 4% formaldehyde, and stood for 24 h. The tissue samples were dehydrated in a series of graded ethanol, and embedded in paraffin after dehydration. After the paraffin was solidified, the intestinal paraffin was cut into slices with a thickness of 5 μm with a microtome, followed by hematoxylin-eosin staining, and encapsulated to make sections. The prepared sections were observed with a Nikon Ni-U microscope imaging system (Nikon Ni-U, Japan), and the intestinal villus height and width, crypt depth, muscular thickness, and the number of goblet cells were counted according to the method described in a previous study (Huang et al., 2022).

Another fish was randomly selected from each cage in the control, Lvs-GG and Hvs-GG groups for dissection, and the hindgut tissue was fixed with 2.5% glutaraldehyde. After 24 h fixation, the tissues were transferred into phosphate-buffered saline containing 2% osmium tetroxide, and then the tissues were dehydrated with a series of graded ethanol. After dehydration, the tissue was embedded in epoxy resin 812 and then cut into ultrathin sections using an ultra-microtome (Leica EM UC7, Japan) for uranyl acetate and lead citrate staining. Finally, the morphology of the intestinal microvilli and intestinal epithelial cells was observed using a transmission electron microscope (HITACHI HT7600, Japan).

### Chemical Analysis

The chemical composition analysis of the experimental diets, whole-body and feces refer to the standard method (AOAC, 2005). The samples were dried at 105°C to constant weight to measure the moisture content, and crude protein was determined using the Kjeldahl method (N × 6.25); crude lipid was determined using the Soxhlet extraction method; crude ash was determined by burning in a muffle furnace at 550°C for 16 h. The viscosity of experimental diets was measured using a viscometer (LV-SSR, Shanghai Fang Rui Instrument Co., Ltd., Shanghai, China). Briefly, the feed was first crushed to pass through an 80 mesh sieve, then 1 g of feed powder was mixed with 10 ml of deionized water, incubated at 25°C for 30 min, followed by centrifugation at 10,000 g for 10 min. Finally, the supernatant was used for viscosity determination.

### Intestinal Enzyme Activity Analysis

Sample preparation: wet intestine tissues were accurately weighted and plus ninefold volume of ice-cold phosphate buffer (PBS, pH 7.4), then homogenized using a homogenizer and centrifuged for 15 min at 3,000 rpm/min. Finally, the supernatant was used for digestive enzyme activity analysis. The activities of intestinal lipase, protease, amylase, creatine kinase (CK), Na^+^/K^+^-ATPase and alkaline phosphatase (AKP) were measured by commercial kits (ELISA, Shanghai Enzyme Link Biotechnology Co., Ltd.) following the kit instructions (No. ML036371, No. ML036449, No. ML652041, No. ML036438, No. ML036470 and No. ML556611, respectively).

### Serum Biochemical Indices Analysis

The concentration of total cholesterol (T-CHO), low-density lipoprotein (LDL-C), high-density lipoprotein (HDL-C), triglyceride (TG), malondialdehyde (MDA) and the activity of superoxide dismutase (SOD), peroxidase (POD), catalase (CAT), aspartate aminotransferase (AST) and alanine aminotransferase (ALT) in the serum were measured using commercially available kits (Nanjing Jiancheng Bioengineering Institute, Nanjing, China) following the kit instructions (No. A111-1-1, No. A113-1-1, No. A112-1-1, No. A003-1-2, No. A110-1-1, No. A001-3-2, No. A084-2-1, No. A007-1-1, No. C010-2-1 and No. C009-2-1, respectively).

### Real-Time Quantitative PCR Assay

Total intestinal RNA was extracted with Trizol kit (Quanjin Biology, Beijing, China), and its purity and concentration were tested. Subsequently, qualified total RNA samples were used as templates to synthesize complementary DNA (cDNA) with a reverse transcription kit (Accurate Biology, China), and stored at −20°C until use. The primer sequences used for fluorescence quantification are shown in Table 2, and the primers were synthesized by Shanghai Sangon Biotech Co., Ltd., (Shanghai, China). The mRNA expression levels were detected using a high-throughput fluorescent quantitative PCR instrument (480II) (Light Cycler480II, Thermo) under a 10 µL SYBR^®^ Green Premix Pro Taq HS qPCR Kit II (Accurate Biology, China) reaction system. Four replicate assays were performed in each sample, and melting curve analysis was performed after each reaction to check product specificity. The mRNA expression of all genes was calculated by the 2^−ΔΔCT^ method, and the mRNA expression level of the target gene was normalized with the *ef1α* mRNA expression of the control group as the standard.

**TABLE 2:** Primer sequences for real-time PCR.

Gene	Primer type	Sequence 5′-3′	E-value (%)	Tm (°C)	Accession no.
Ef1α	F^a^	TGC​TGC​TGG​TGT​TGG​TGA​GTT	97.99	61.2	XM_038724777.1
	R^a^	TTC​TGG​CTG​TAA​GGG​GGC​TC			
IGF1	F	CTT​CAA​GAG​TGC​GAT​GTG​C	97.96	59.3	XM_038736342.1
	R	GCC​ATA​GCC​TGT​TGG​TTT​ACT​G			
GLP-2	F	CCG​AGC​AAC​ACT​GGT​ACT​GA	115.63	56.6	XM_038738328.1
	R	GCT​GAG​AGT​GAG​GTT​GAC​GA			

F^a^, forward primer; R^a^, reverse primer.

IGF1, insulin-like growth factors -1; GLP-2, Glucagon-like peptide 2.

### Statistical Analysis

Data in this study were presented as mean ± standard error (Means ± SEM). All data were subjected to one-way analysis of variance (ANOVA) with SPSS (v22.0) software, and Turkey’s multiple range test was performed when the difference was significant. *p* < 0.05 represents significant differences.

## Results

### Growth Performance and Feed Utilization

The survival rate (SR) of juvenile largemouth bass was not significantly affected by the experimental diets (*p* < 0.05; Table 3). The final body weight (FBW), weight gain rate (WGR), specific growth rate (SGR), protein efficiency ratio (PER), protein deposition rate (PDR) and lipid deposition rate (LDR) in the guar gum groups were significantly lower than those in the control group; these parameters were also significantly decreased with increased guar gum viscous (*p* < 0.05). Conversely, the feed intake (FI) and feed conversion ratio (FCR) in the guar gum groups were significantly higher than those in the control group; these parameters were also increased dramatically with increased guar gum viscous (*p* < 0.05).

**TABLE 3:** Effects of different viscous guar gum on growth performance and feed utilization of juvenile largemouth bass.

Group	Control	Lvs-GG	Mvs-GG	Hvs-GG
FBW (g)	67.23 ± 1.26^c^	56.40 ± 1.42^b^	49.60 ± 0.97^a^	50.96 ± 1.49^a^
SR^a^ (%)	98.75 ± 1.25	98.13 ± 1.20	99.38 ± 0.63	99.38 ± 0.63
WGR^b^ (%)	1118.50 ± 20.53^c^	942.42 ± 24.59^b^	852.14 ± 28.32^a^	848.99 ± 24.87^a^
SGR^c^ (%/d)	4.31 ± 0.03^c^	4.00 ± 0.05^b^	3.82 ± 0.06^a^	3.82 ± 0.05^a^
FI^d^ (% BW/d)	2.85 ± 0.05^a^	3.34 ± 0.08^b^	3.69 ± 0.14^c^	3.65 ± 0.09^c^
FCR^e^	0.95 ± 0.02^a^	1.16 ± 0.03^b^	1.31 ± 0.06^c^	1.30 ± 0.04^c^
PER^f^	2.46 ± 0.05^c^	2.04 ± 0.06^b^	1.74 ± 0.06^a^	1.82 ± 0.06^a^
PDR^g^ (%)	38.57 ± 0.77^c^	31.42 ± 0.89^b^	27.21 ± 0.93^b^	27.53 ± 0.92^a^
LDR^h^ (%)	67.91 ± 1.29^c^	44.41 ± 1.21^b^	31.87 ± 1.08^b^	29.57 ± 0.97^a^

FBW, final body weight; SR, survival rate; WGR, weight gain rate; SGR, specific growth rate; FI, feed intake; FCR, feed conversion ratio; PER, protein efficiency ratio; PDR, protein deposition rate; LDR, lipid deposition rate.

Values are presented as means ± S.E.M (*n* = 4). Different superscript letters in the same row means there was a significant difference between data (*p* < 0.05).

a SR (%) = 100 × the final fish number/the initial fish number.

b WGR (%) = 100 × (final body weight—initial body weight)/initial body weight.

c SGR (%/d) = 100 × ((ln final body weight)—(ln initial body weight))/d.

d FI (% BW/d) = 100 × final body weight/[(final body weight + initial body weight)/2 × d].

e FCR, total feed intake (dry matter)/(final biomass—initial biomass + biomass of dead fish).

f PER = (final body weight—initial body weight)/total protein intake.

g PDR (%) = 100 × (final body weight × final body protein—initial body weight × initial body protein)/total protein intake.

h LDR (%) = 100 ×final body weight × final body lipid—initial body weight × initial body lipid)/total lipid intake.

### Morphological Parameters

The condition factors (CF), organ index (OI), and the moisture, crude protein and ash concentrations of juvenile largemouth bass were not significantly affected by the experimental diets (*p* > 0.05; Table 4). The hepasomatic index (HSI) in the guar gum groups was considerably lower than that in the control group, and this parameter was markedly decreased with increased guar gum viscous (*p* < 0.05). The viserosomatic index (VSI) and intestinal length index (ILI) in the guar gum groups were significantly higher than that in the control group, and the VSI was increased dramatically with increased guar gum viscous (*p* < 0.05). The crude lipid concentration in the guar gum groups was considerably lower than that in the control group, and decreased substantially with increased guar gum viscous (*p* < 0.05).

**TABLE 4:** Effects of different viscous guar gum on morphological parameters of juvenile largemouth bass.

Group	Control	Lvs-GG	Mvs-GG	Hvs-GG
Morphological parameters				
CF^a^ (g/cm^3^)	2.20 ± 0.04	2.12 ± 0.04	2.12 ± 0.03	2.10 ± 0.03
OI^b^ (%)	8.04 ± 0.13^b^	7.64 ± 0.12^a^ ^,^ ^b^	7.60 ± 0.22^a^ ^,^ ^b^	7.39 ± 0.12^a^
HSI^c^ (%)	1.86 ± 0.06^c^	0.98 ± 0.08^b^	0.86 ± 0.04^a^ ^,^ ^b^	0.76 ± 0.03^a^
VSI^d^ (%)	0.69 ± 0.02^a^	1.09 ± 0.03^b^	1.21 ± 0.03^c^	1.26 ± 0.03^d^
ILI^e^ (%)	0.86 ± 0.01^a^	0.96 ± 0.14^b^	0.94 ± 0.01^b^	0.95 ± 0.02^b^
Body composition, %				
Moisture	72.01 ± 1.06	72.59 ± 0.92	72.70 ± 1.12	73.73 ± 1.27
Crude protein	15.66 ± 0.20	15.29 ± 0.18	15.73 ± 0.17	15.79 ± 0.04
Crude lipid	8.53 ± 0.09^c^	7.39 ± 0.08^b^	7.33 ± 0.20^b^	6.68 ± 0.03^a^
Ash	4.06 ± 0.20	3.97 ± 0.12	4.10 ± 0.15	4.10 ± 0.12

CF, condition factor; OI: organ index; HSI, hepasomatic index; VSI, viserosomatic index; ILI, intestinal length index.

Values are presented as means ± S.E.M (*n* = 4). Different superscript letters in the same row means there was a significant difference between data (*p* < 0.05).

a CF (g/cm^3^) = body weight/(body length)^3^.

b OI (%) = 100 × visceral weight/body weight.

c HSI (%) = 100 × hepatic weight/body weight.

d VSI (%) = 100 × intestinal weight/body weight.

e ILI (%) = 100 × intestinal length/body length.

### Apparent Digestibility

The apparent digestibility of dry matter, crude protein and crude lipid in the guar gum groups were considerably lower than those in the control group, and these parameters decreased substantially with increased guar gum viscous (*p* < 0.05; Table 5).

**TABLE 5:** Effects of different viscous guar gum on nutrient apparent digestibility of juvenile largemouth bass.

Group	Control	Lvs-GG	Mvs-GG	Hvs-GG
Dry matter (%)	85.52 ± 1.24^c^	80.32 ± 0.60^b^	74.42 ± 0.26^a^	73.98 ± 0.45^a^
Crude protein (%)	91.32 ± 0.36^c^	84.36 ± 1.07^b^	80.10 ± 0.25^a^	80.65 ± 0.75^a^
Crude lipid (%)	90.88 ± 0.2^c^	82.66 ± 0.48^b^	71.54 ± 0.40^a^	72.05 ± 0.56^a^

Values are presented as means ± S.E.M (*n* = 4). Different superscript letters in the same row means there was a significant difference between data (*p* < 0.05).

Apparent digestibility coefficient of dry matter (%) = 100 × [1– (dietary Y_2_O_3_ level/feces Y_2_O_3_ level)].

Apparent digestibility coefficient of nutrients (%) = 100 × [1– (dietary Y_2_O_3_ level/feces Y_2_O_3_ level) × (feces nutrient level/dietary nutrient level)].

### Intestinal Digestive and Absorptive Enzyme Activity

Intestinal amylase activity was not significantly affected by the experimental diets (*p* < 0.05; Table 6). Intestinal protease, lipase and creatine kinase (CK) activities in the guar gum groups were significantly lower than those in the control group, and intestinal protease and lipase activities decreased considerably with increased guar gum viscous (*p* < 0.05). Intestinal alkaline phosphatase (AKP) activity in the Hvs-GG group was considerably lower than that in the Lvs-GG and control groups (*p* < 0.05). Intestinal Na^+^/K^+^-ATPase activity in the Mvs-GG and Hvs-GG groups was considerably lower than in the Lvs-GG and control groups (*p* < 0.05).

**TABLE 6:** Effects of different viscous guar gum on intestinal digestive and absorptive enzyme activities.

Group	Control	Lvs-GG	Mvs-GG	Hvs-GG
Protease (U/g protein)	4.55 ± 0.14^c^	4.09 ± 0.13^b^	3.53 ± 0.26^a^	3.37 ± 0.17^a^
Lipase (U/g protein)	0.85 ± 0.04^c^	0.77 ± 0.05^bc^	0.71 ± 0.03^b^	0.53 ± 0.02^a^
Amylase (U/g protein)	0.33 ± 0.04	0.35 ± 0.04	0.32 ± 0.03	0.28 ± 0.03
Creatine kinase (U/mg protein)	0.16 ± 0.02^b^	0.12 ± 0.02^a^	0.12 ± 0.01^a^	0.11 ± 0.01^a^
Na^+^/K^+^-ATPase (U/mg protein)	24.37 ± 1.44^b^	22.54 ± 0.72^b^	22.11 ± 0.81^ab^	19.42 ± 0.37^a^
Alkaline phosphatase (U/g protein)	145.63 ± 5.69^b^	158.22 ± 5.29^b^	133.04 ± 3.01^a^	132.11 ± 5.01^a^

Values are presented as means ± S.E.M (*n* = 4). Different superscript letters in the same row means there was a significant difference between data (*p* < 0.05).

### Serum Biochemical Parameters

Serum LDL-C concentration and AST, CAT and POD activities were not significantly affected by the experimental diets (*p* > 0.05; Table 7). Serum HDL-C, T-CHO, TG) concentrations and SOD activity in the guar gum groups were significantly lower than those in the control group, and serum TG concentration decreased considerably with increased guar gum viscous (*p* < 0.05). Serum MDA concentration in the Mvs-GG and Hvs-GG groups was significantly higher than that in the Lvs-GG and control groups (*p* < 0.05). Serum ALT activity in the Hvs-GG group was considerably higher than that in other groups (*p* < 0.05).

**TABLE 7:** Effects of different viscous guar gum on serum biochemical parameters of juvenile largemouth bass.

Group	Control	Lvs-GG	Mvs-GG	Hvs-GG
HDL-C (mmol/L)	5.11 ± 0.63^b^	3.66 ± 0.37^a^	3.50 ± 0.23^a^	3.40 ± 0.19^a^
LDL-C (mmol/L)	3.53 ± 0.23	3.24 ± 0.34	3.26 ± 0.06	3.26 ± 0.41
T-CHO (mmol/L)	10.75 ± 0.60^b^	7.41 ± 0.54^a^	7.26 ± 0.29^a^	7.53 ± 0.50^a^
TG (mmol/L)	10.17 ± 0.75^c^	8.88 ± 0.34^b^	7.18 ± 0.17^a^	6.94 ± 0.23^a^
ALT (U/L)	3.84 ± 0.15^a^	4.40 ± 0.42^a^	3.77 ± 0.37^a^	6.64 ± 0.34^b^
AST (U/L)	15.75 ± 0.40	15.68 ± 1.09	14.90 ± 1.84	15.79 ± 0.97
SOD (U/mL)	217.72 ± 10.52^b^	174.30 ± 6.55^a^	180.47 ± 5.28^a^	169.16 ± 6.50^a^
MDA (nmol/ml)	19.23 ± 1.20^a^	19.37 ± 1.62^a^	35.14 ± 1.56^b^	34.23 ± 0.90^b^
CAT(U/ml)	6.23 ± 0.26	6.61 ± 0.25	6.46 ± 0.28	6.79 ± 0.25
POD (U/ml)	1.31 ± 0.04	1.29 ± 0.04	1.25 ± 0.03	1.30 ± 0.03

HDL-C, high-density lipoprotein; LDL-C, low-density lipoprotein; T-CHO, total cholesterol; TG, triglyceride; MDA, malondialdehyde: ALT, alanine aminotransferase; AST, aspartate aminotransferase; POD, peroxidase; CAT, catalase; SOD, superoxide dismutase.

Values are presented as means ± S.E.M (*n* = 4). Different superscript letters in the same row means there was a significant difference between data (*p* < 0.05).

### Intestinal Morphology

**FIGURE 1:**
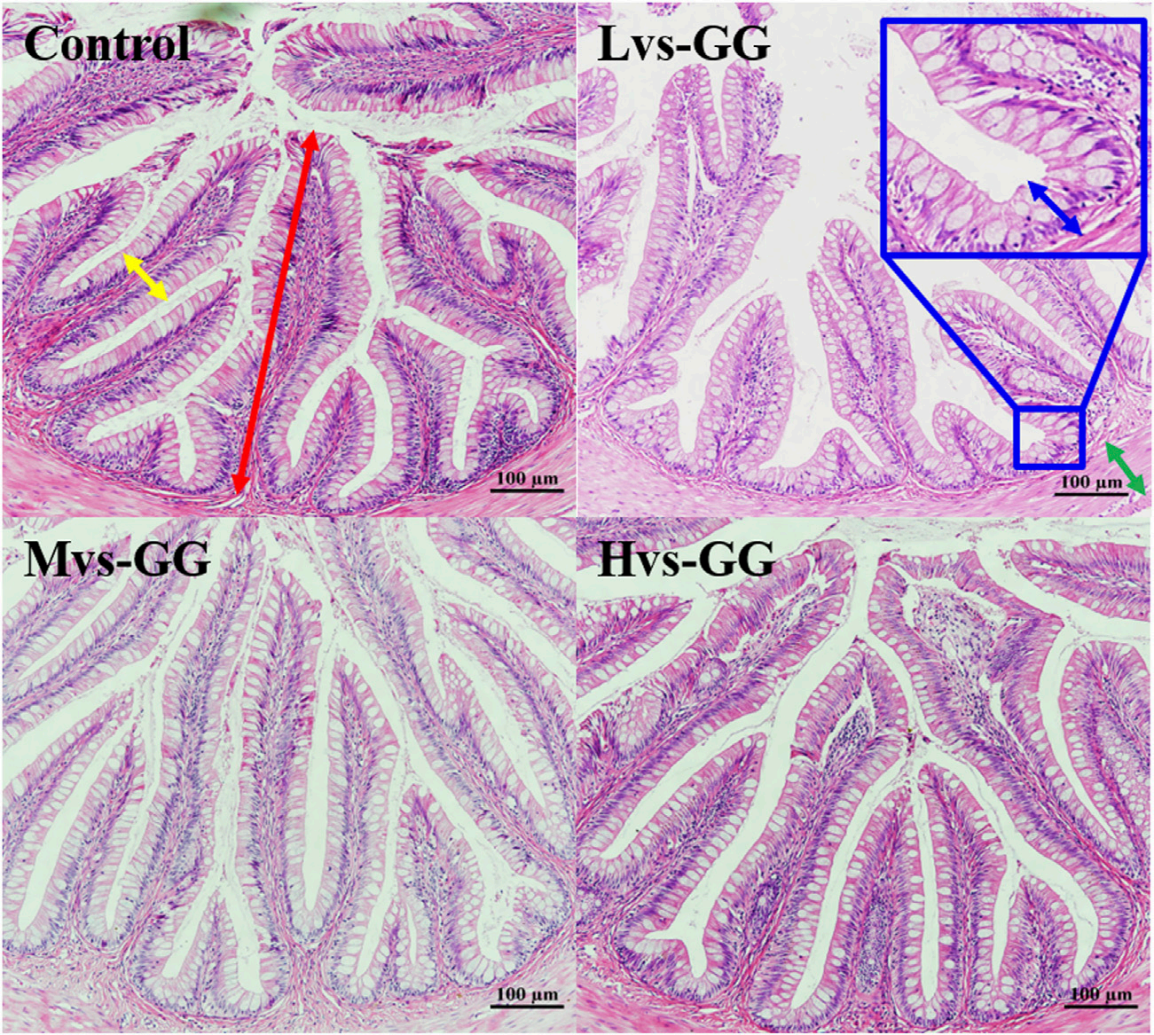
Effects of increasing dietary guar gum viscous on hindgut morphology of juvenile largemouth bass (H&E staining, magnification ×200). Yellow double-side arrow: villus width; Red double-side arrow: villus height; green double-side arrow: muscularis thickness; blue double-side arrow: crypt depth; blue arrow: goblet cell.

**FIGURE 2:**
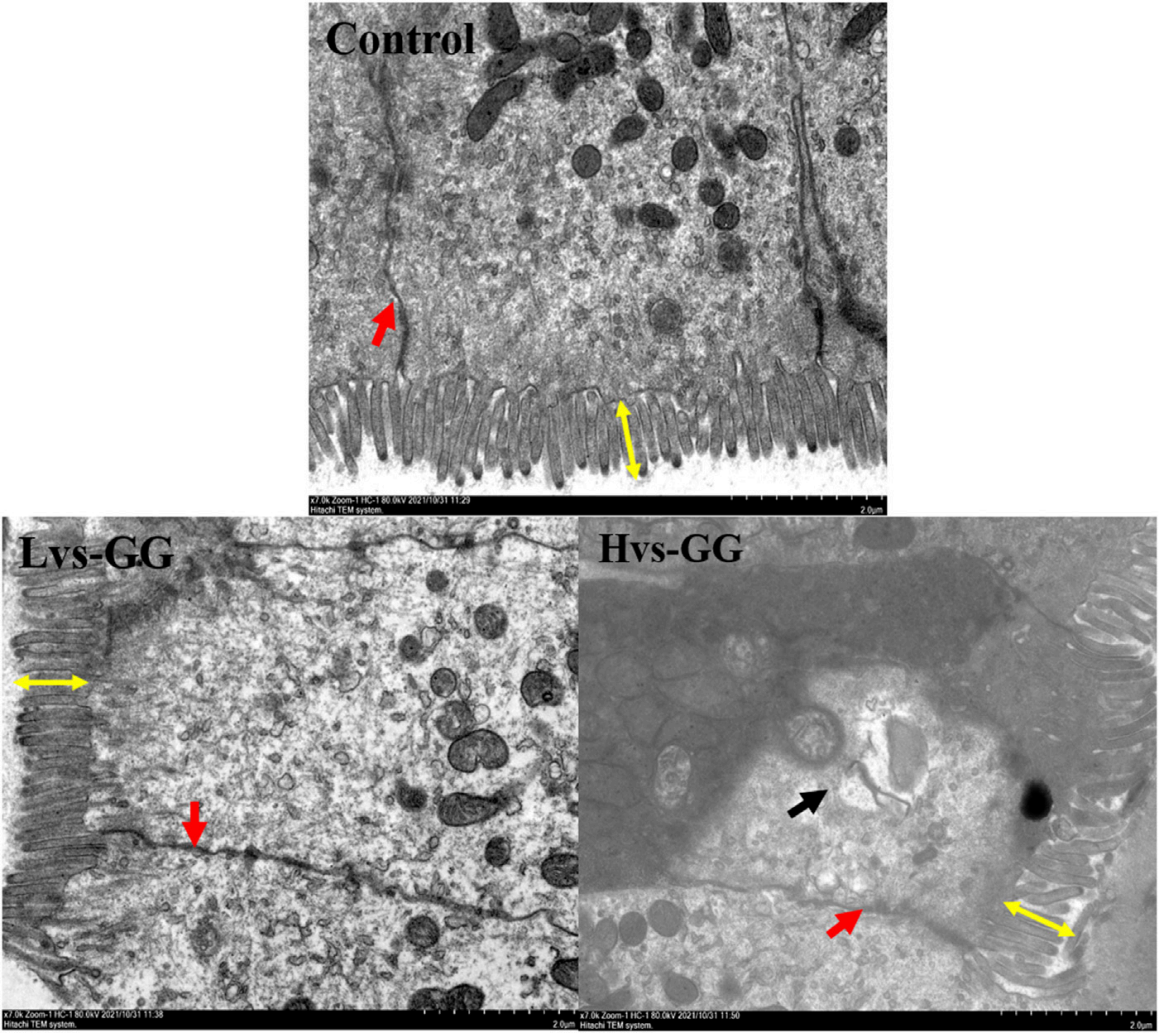
Effects of increasing dietary guar gum viscous on hindgut morphology of juvenile largemouth bass (Transmission electron microscope observation, magnification ×7,000). Yellow double-side arrow: microvillus height; black arrow: epithelial cell death; red arrow: epithelial cell interval.

The morphological observations of the hindgut are shown in Figure 1 and Figure 2. Intestinal villus width and crypt depth were not significantly affected by the experimental diets (*p* > 0.05; Table 8). Intestinal villus height and muscular thickness in the guar gum groups were considerably higher than those in the control group (*p* < 0.05). The goblet cell relative number in the Mvs-GG and Hvs-GG groups was considerably lower than in the Lvs-GG and control groups (*p* < 0.05). The microvillus height in the Lvs-GG and Hvs-GG groups was considerably lower than in the control group (*p* < 0.05).

**TABLE 8:** Effects of different viscous guar gum on hindgut morphology of juvenile largemouth bass.

Group	Control	Lvs-GG	Mvs-GG	Hvs-GG
Villus height (μm)	518.35 ± 24.40^a^	646.97 ± 18.27^b^	625.79 ± 26.57^b^	629.12 ± 22.00^b^
Villus width (μm)	103.45 ± 13.28	98.47 ± 5.42	99.60 ± 10.54	107.94 ± 7.05
crypt depth (μm)	25.59 ± 3.14	23.69 ± 1.62	23.26 ± 1.58	23.89 ± 1.50
Muscularis thickness (μm)	110.06 ± 6.48^a^	132.71 ± 6.07^b^	147.71 ± 7.88^b^	133.82 ± 4.45^b^
Goblet cell relative number (per 100 μm)	17.00 ± 0.50^b^	16.29 ± 0.72^b^	12.50 ± 0.65^a^	13.20 ± 0.49^a^
Microvillus height (μm)	1.31 ± 0.03^b^	1.16 ± 0.04^a^	-	1.22 ± 0.05^a^

Values are presented as means ± S.E.M (*n* = 4). Different superscript letters in the same row means there was a significant difference between data (*p* < 0.05).

### Intestinal Development-Related Gene Expression

**FIGURE 3:**
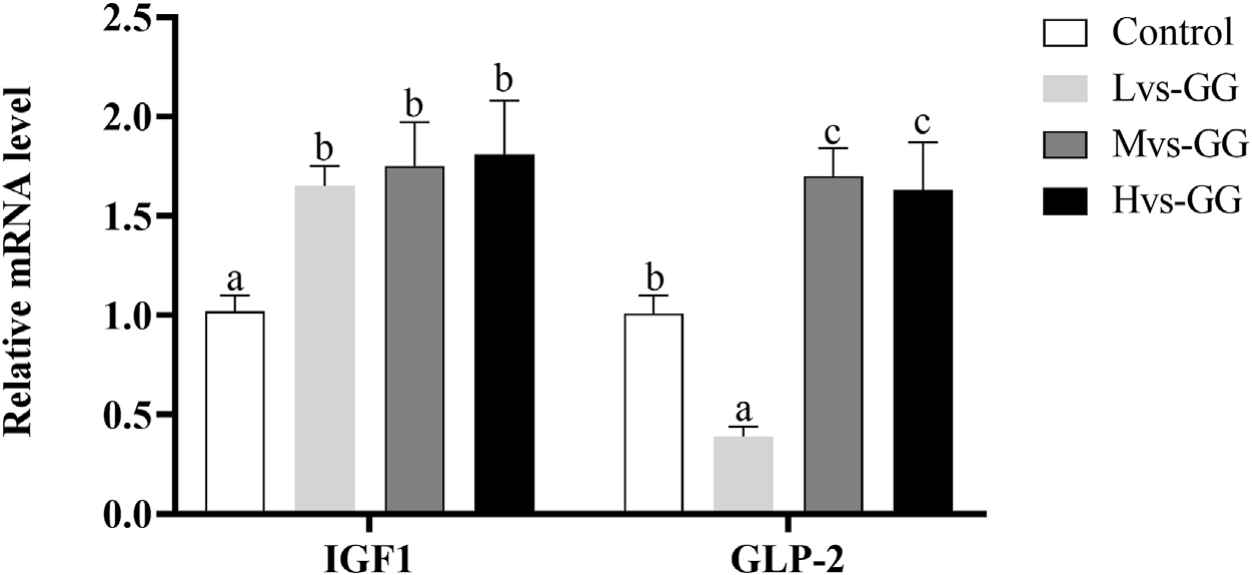
Effects of increasing dietary guar gum viscous on the expression levels of intestinal IGF1 and GLP-2 in juvenile largemouth bass fed different viscous guar gum. Bars represent the mean ± SEM (*n* = 4), and different superscript letters represent significant differences between treatments (*p* < 0.05).

The expression level of GLP-2 in the Lvs-GG group was significantly lower than that in the control group, while increased significantly in the Mvs-GG and Hvs-GG groups (*p* < 0.05; Figure 3). The expression level of IGF-1 in the guar gum groups were significantly higher than that in the control group (*p* < 0.05).

## Discussion

This study was the first attempt to evaluate the effects of different viscous guar gum on growth, apparent nutrient digestibility, intestinal development and morphology in largemouth bass. In early studies, guar gum has been reported to have the ability to lower serum cholesterol concentrations, control body weight and alleviate obesity in human (Jenkins et al., 1975; Tuomilehto et al., 1980; Casas et al., 2000; Butt et al., 2007). Although these features may benefit humans, for farmed animals, such as fish, it means a decrease in growth performance, which is detrimental to farming profitability. Recently, guar gum (galactomannan) has attracted increasing attention as an anti-nutritional factor or feed binder in fish (Brinker, 2009; Brinker and Friedrich, 2012; Thombare et al., 2016; Gao et al., 2019), and several studies have shown that dietary supplementation with guar gum is beneficial for improving feed quality but not for fish growth (Tran-Tu et al., 2018; Gao et al., 2019). In the present study, dietary guar gum significantly inhibited the growth performance of largemouth bass, and fish fed with high viscous guar gum had the poorest growth performance, suggesting that high viscous guar gum is more detrimental to the growth of juvenile largemouth bass. In a previous study, increasing dietary guar gum levels also decreased the growth performance of gibel carp (*Carassius gibelio*) due to increased dietary guar gum levels increased dietary viscous (Gao et al., 2019). This evidence demonstrated that viscous is the prominent anti-nutritional feature of guar gum, and high viscous diets are detrimental to fish growth. Generally, increasing dietary viscous delays intestinal evacuation and thus adversely affects feed intake (Leenhouwers et al., 2006; Sinha et al., 2011). In a previous study, increasing dietary guar gum levels from 1% to 5% resulted in a significantly decreased FI of gibel carp (Gao et al., 2019). However, changes in FI in this study showed that increasing dietary viscosity promotes fish feeding. Similarly, the FI of rainbow trout (*Oncorhynchus mykiss*) fed SNSP (viscous) diets was significantly increased compared with fish fed a normal diet (Deng et al., 2021). This evidence suggests that the effects of increasing dietary viscosity on FI in fish remain controversial, and the difference may be species-related, which requires further study.

Digestive and absorptive enzymes are essential for fish to utilize feed nutrients and play an important role in fish growth (Li et al., 2019; Lin S et al., 2020). Also, the activity of digestive and absorptive enzymes determines the digestibility and utilization of feed nutrients by fish. Meanwhile, the characteristics and quantities of dietary components will inevitably affect the activity of digestive and absorptive enzymes in fish (Hu et al., 2018; Zhang et al., 2021; Mardones et al., 2022). In a previous study, high dietary viscous has been reported to inhibit feed utilization by altering the mixing process between digestive enzymes and substrates, or hindering the effective interaction of digestive enzymes on the intestinal mucosal surface (Sinha et al., 2011). In this study, the activity of protease, lipase, CK, Na^+^/K^+^-ATPase and AKP were all shown to have a decreased trend with increased dietary guar gum viscous, and most of these parameters were lowest in the Hvs-GG group, indicating that high viscous guar gum is more detrimental to the digestive and absorptive enzyme activities in juvenile largemouth bass. Similarly, increasing dietary viscosity decreased the activity of chymotrypsin and AKP in gibel carp (Gao et al., 2019), and reduced the intestinal trypsin activity in rainbow trout (Deng et al., 2021). This evidence suggest that high viscous diets may also inhibit the utilization of feed nutrients on fish by directly reducing intestinal digestive and absorptive enzyme activities. Although the nutrient content of feces may be lost through contact with water, making it impossible to provide precise nutrient digestibility of the experimental diet in this study, the near-visual values of nutrient digestibility are still valid to illustrate the effect of different viscous guar gum on nutrient digestibility. In this study, we noticed that a significant decrease in apparent nutrient digestibility with increasing guar gum viscosity, which is consistent with a decrease in intestinal digestive and absorptive enzyme activity. Therefore, the decrease in digestive and absorptive enzyme activities may reasonably explain the reduction of apparent digestibility of dry matter, crude protein and crude lipid in fish fed on guar gum diets. Furthermore, an increase in dietary viscosity has been reported to cause mucin efflux with the digesta, resulting in endogenous nitrogen loss and reduced apparent protein digestibility (Piel et al., 2005; Sinha et al., 2011). Hence, the significant reduction of apparent protein digestibility in fish fed guar gum diets may also be associated with endogenous nitrogen loss.

Previous studies have confirmed that the structure of guar gum (galactomannan) has a large number of hydroxyl units (Ebringerová, 2005). These hydroxyl units can bind to mineral elements, causing mineral element loss. In fish, several studies have demonstrated that dietary NSPs inhibit the utilization of mineral elements (e.g., Na, K, Zn, Mg, Ca, P, Cu, and Fe), as well as an increase in the mineral elements’ concentration of the feces (Leenhouwers et al., 2007a; Leenhouwers et al., 2007b; Hansen and Storebakken, 2007). Noteworthy, some mineral elements are essential for the activity of digestive and absorptive enzyme activities. For instance, Ca act as an activator of proteases (Li et al., 2017), and Na and P as substrates can effectively regulate Na^+^/K^+^-ATPase activity (Gal-Garber et al., 2003). Therefore, the decrease in digestive and absorptive enzyme activities in fish fed guar gum diets may also be associated with the loss of mineral elements.

It is well known that the liver and gastrointestinal tract constitute the primary digestive system of fish, where dietary nutrients are absorbed by the intestine and then transferred to the liver for storage or utilization. Therefore, fish slaughter parameters (OI, HSI, VSI and ILI) are commonly used to assess nutritional status (Knutsen et al., 2019; Shen et al., 2021). Generally, HSI can effectively reflect the accumulation of nutrients, while VSI and ILI were assumed to evaluate the status of digestion and absorption abilities in fish. In this study, HSI was significantly decreased in fish fed guar gum diets, and this parameter was significantly reduced with increased dietary guar gum viscous. The decrease in HSI may be associated with poor apparent digestibility of feed nutrients, due to the reduced nutrient intake reduce the accumulation of nutrients in the liver. In previous studies, increasing dietary SNSP levels has been reported to increase the length and size of digestive organs in monogastric animals (including fish), due to the increased viscosity (Sinha et al., 2011). In this study, VSI and ILI were also significantly increased in fish fed guar gum diets, accompanied by a significant upregulation of intestinal development-related genes, including IGF1 and GLP-2. These results suggest that increasing dietary viscosity may prolong intestinal length via upregulation of intestinal development-related genes, but the mechanism remains unclear. Similarly, dietary supplementation with guar gum significantly increased the gastrointestinal tract weight of African catfish (*Clarias gariepinus*) (Leenhouwers et al., 2006). Combined with lower digestive and absorptive enzyme activities and poor apparent nutrient digestibility, it can be speculated that prolonging intestinal length may be a strategy for juvenile largemouth bass to cope with high viscous guar gum diets, by increasing digestion and absorption area to obtain more nutrients. Moreover, poor lipid digestibility ultimately leads to a significant reduction in serum HDL-C, T-CHO, TG and whole-body crude lipid concentrations in fish fed guar gum diets.

AST and ALT are enzymes involved in amino acid metabolism and are mainly distributed in hepatocytes, released into the blood when hepatocytes are damaged. Therefore, the activity of AST and ALT are commonly used to assess the liver health status of fish (Deng et al., 2017). In the present study, changes in ALT activity indicate that high viscous guar gum induced liver damage in juvenile largemouth bass. In addition, fish fed high viscous guar gum diets also showed a significant decrease in serum SOD activity and an increase in MDA concentration, suggesting that high viscous guar gum reduces the antioxidant capacity of juvenile largemouth bass. More importantly, a decrease in antioxidant capacity usually leads to increased radicals, thereby inducing oxidative damage (Lin et al., 2018). Hence, liver damage caused by a high viscous diet may be associated with decreased antioxidant capacity. Similarly, high SNSP diets also induced liver impairment in yellow catfish (*Pelteobagrus fulvidraco*) (Cai et al., 2019).

Dietary components inevitably affect the intestinal morphology of fish; hence, intestinal morphology is commonly used to evaluate the effects of diets on fish intestines (Hartviksen et al., 2014; Huang et al., 2022). More importantly, the intestinal physiological functions are closely associated with the morphology (Fang et al., 2019; Li et al., 2020). For instance, changes in villus height, folds and goblet cell numbers can affect the digestive and absorptive functions of the intestine (Lin A et al., 2020; Liu et al., 2021). In general, factors that increase the digestive and absorption area are conducive to promote digestion and absorption in the intestinal tract. In this study, the microvillus height was significantly decreased in fish fed guar gum diets, suggesting that increasing dietary viscosity is detrimental to intestinal digestive and absorption functions. Conversely, the villus height and muscular thickness were significantly increased in fish fed guar gum diets. Combined with poor nutrient digestibility, we tend to believe that the increase in villus height is an adaptation to the adverse effects of high viscous diets. Intestinal muscular thickness can represent intestinal peristaltic capacity (Huang et al., 2022). Increasing dietary viscous has been associated with increased digesta viscous Leenhouwers et al., 2007a), delayed gastric emptying and decreased intestinal internal oxygen tension, which providing high-quality conditions for anaerobic microbial proliferation (Choct, 1997; Sinha et al., 2011). More importantly, the expansion of some anaerobic microbial may increase the concentrations of toxic metabolites (Carré et al., 1995), which are detrimental to intestinal health. In this case, increased intestinal motility may provide benefits in alleviate the negative effects of high viscous diets. Thus, it can be speculated the increase of intestinal muscularis thickness is an adaptive development of high viscous diets.

## Conclusion

In conclusion, guar gum diets adversely affected intestinal morphology, decreased intestinal digestive and absorptive enzyme activities, and caused poor nutrient digestibility and growth performance in juvenile largemouth bass. Moreover, the adverse effects of guar gum are closely related to its viscous, and high viscous guar gum produces more extreme negative impacts on juvenile largemouth bass. Therefore, the application of guar gum as an aquafeed binder requires consideration of its viscous.

## Data Availability

The original contributions presented in the study are included in the article/supplementary materials, further inquiries can be directed to the corresponding authors.
